# Supplementary material for: Functional brain connectomes reflect acute and chronic cannabis use
Source: Sci Rep. 2022 Feb 14;12:2449. doi: 10.1038/s41598-022-06509-9 (PMC8844352; doi:10.1038/s41598-022-06509-9)
Supplement: Supplementary file 1 — Supplementary Information. [file 41598_2022_6509_MOESM1_ESM.docx]

**Functional brain connectomes reflect acute and chronic cannabis use.**

**Supplementary information**

Ramaekers JG ^1^, Mason NL^1^, Toennes SW^2^, Theunissen EL^1^, Amico E^3,4^

^1^Faculty of Psychology and Neuroscience, Maastricht University, The Netherlands

^2^Institute of Legal Medicine, University of Frankfurt, Germany

^3^Institute of Bioengineering, Center for Neuroprosthetics, EPFL, Geneva, Switzerland

^4^Department of Radiology and Medical Informatics, University of Geneva, Switzerland

Correspondence: [j.ramaekers@maastrichtuniversity.nl](mailto:j.ramaekers@maastrichtuniversity.nl) and [enrico.amico@epfl.ch](mailto:enrico.amico@epfl.ch)

**
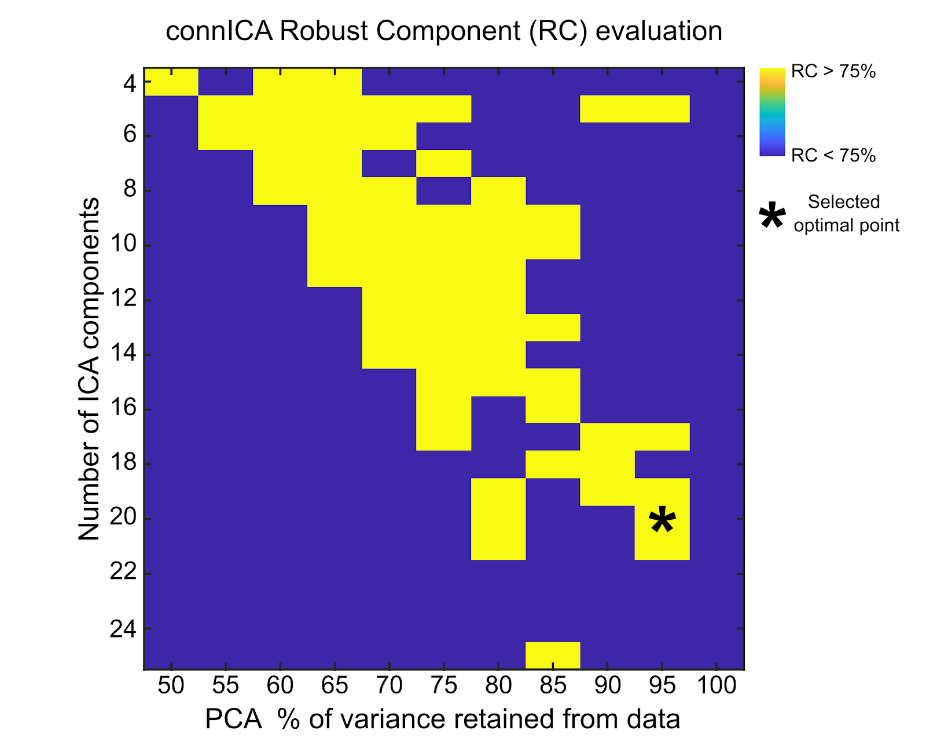
**

**Fig. S1. Exploration of optimal connICA parameters.**The two free parameters of the connICA methodology ^1^, i.e. the number of ICA components and the percentage of variance retained, were explored to maximize: 1) the number of robust components and, 2) the percentage of variance retained from the data. The optimal point at 95% retained variance in PCA and 20 independent components is indicated by an asterisk.

**
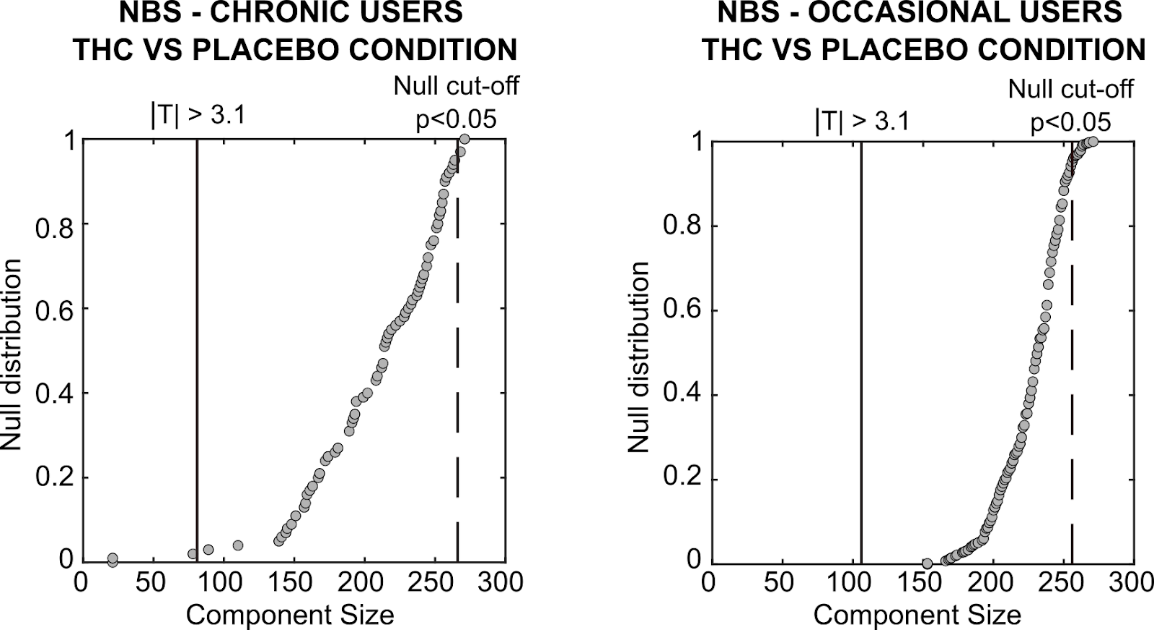
**

**Fig. S2. NBS analysis of THC vs placebo effects in each cannabis user group.** Giant component size extracted from the T-statistic matrix obtained when comparing THC vs placebo conditions within the cannabis user groups. Note how the effect does not survive the cut-off threshold obtained by random shuffling the drug labels, in both groups

| **Table S1.** Mean subject characteristics (SD) and history of drug use for occasional and chronic cannabis users who completed the study (N=26). | | | | | |
| --- | --- | --- | --- | --- | --- |
| **Variable** | **Occasional Users** | **Chronic Users** | **Value** | **df** | ***P* value** |
| Gender (male/female), n, total | 7/7, 14 | 9/3, 12 | χ2 = 1.71^‡^ | 1 | 0.19 |
| Age, years | 22.14 (2.51) | 21.83 (2.25) | *t*=0.33^†^ | 24 | 0.74 |
| History of cannabis use, years | 5.21 (2.61) | 5.33 (1.78) | *t*=.-0.13^†^ | 24 | 0.89 |
| Frequency of cannabis use, per week | 1.12 (0.78) | 6.63 (1.40) | *t*=-12.64^†^ | 24 | 0.00* |
| Alcohol consumption, glasses per week | 6.68 (6.68) | 3.17 (2.32) | *t*=1.73^†^ | 24 | 0.10 |
| Caffeine consumption (per week) | 8.07 (7.11) | 8.88 (6.05) | *t*=-0.31^†^ | 24 | 0.76 |
| Nicotine consumption, per week | 14.00 (21.15) | 19.29 (28.23) | *t*=-1.58^†^ | 24 | 0.13 |
| Occasional use of other drugs, n | 9 | 9 | χ2 = 0.00^‡^ | 1 | 1.00 |
| *Significant *P* values  ^†^Independent *t* test  ^‡^χ2 test for frequency data | | | | | |

**Table S2** Mean (SE) subjective high, lapses of attention averaged over two time points in each treatment condition. OU=occasional users; CU=chronic users.

|  | **Subjective High (cm)** | | | **Lapses of attention (#)** | | |
| --- | --- | --- | --- | --- | --- | --- |
|  | Post-resting state 1  (20 min) | Post-resting state 2  (42 min) | average | Pre-resting state 1  (1 min) | Pre-resting state 2  (22 min) | Average |
| **OU** |  |  |  |  |  |  |
| Placebo | 1.21 (.38) | 0.92 (.37) | 1.07 (.31) | 3.50 (.81) | 5.35 (1.51) | 4.42 (1.08) |
| THC | 5.36 (.70) | 3.92 (.61) | 4.73 (.61) | 5.0 (1.26) | 8.07 (1.78) | 6.15 (1.38) |
| **CU** |  |  |  |  |  |  |
| Placebo | 2.50 (.62) | 2.08 (.62) | 2.29 (.57) | 1.25 (.30) | 1.08 (.31) | 1.16 (.23) |
| THC | 4.83 (.75) | 4.08 (.73) | 4.45 (.71) | 1.83 (.34) | 1.91 (.62) | 1.87 (.35) |

**Table S3** Mean (SE) THC, THC-COOH and 11-OH-COOH concentrations at baseline and two time points prior to resting state fMRI in THC condition. OU=occasional users; CU=chronic users.

|  | **THC**  **(ng/ml)** | **THC-COOH**  **(ng/ml)** | **11-OH-THC**  **(ng/ml)** |
| --- | --- | --- | --- |
| **OU** |  |  |  |
| Baseline | 0 | 1.54 (.61) | 0 |
| 6 min (pre-resting state 1) | 8.61 (1.46) | 8.31 (1.78) | 1.61 (.31) |
| 28 min (pre-resting state 2) | 2.88 ((0.50) | 7.78 (1.58) | .93 (.19) |
| **CU** |  |  |  |
| Baseline | 3.48 (.89) | 47.44 (14.33) | 1.55 (.38) |
| 6 min (pre-resting state 1) | 15.86 (3.48) | 48.81 (14.19) | 3.84 (1.13) |
| 28 min (pre-resting state 2) | 6.66 (1.55) | 45.53 (15.43) | 2.10 (.62) |
